# Dr. Eugene F. Cordell

**Published:** 1881-05

**Authors:** 


DR. EUGENE F. CORDELL Has recently been associated with Dr. T. A. Ashby in the editorial conduct of the *Maryland Medical Journal.* Dr. Cordell is quite well known to the profession as a correspondent and contributor to several medical journals, and is a graceful and fluent writer. We welcome him as a co-laborer in the journalistic field, and wish for him, and the enterprise with which he is associated, an extended field of usefulness and honor.

